# Variability in mRNA translation: a random matrix theory approach

**DOI:** 10.1038/s41598-021-84738-0

**Published:** 2021-03-05

**Authors:** Michael Margaliot, Wasim Huleihel, Tamir Tuller

**Affiliations:** 1 Department of Electrical Engineering-Systems, Faculty of Engineering, Tel Aviv University, 69978 Tel Aviv, Israel; 2 Department of Biomedical Engineering, Faculty of Engineering, Tel Aviv University, 69978 Tel Aviv, Israel

**Keywords:** Systems biology, Mathematics and computing, Biomedical engineering

## Abstract

The rate of mRNA translation depends on the initiation, elongation, and termination rates of ribosomes along the mRNA. These rates depend on many “local” factors like the abundance of free ribosomes and tRNA molecules in the vicinity of the mRNA molecule. All these factors are stochastic and their experimental measurements are also noisy. An important question is how protein production in the cell is affected by this considerable variability. We develop a new theoretical framework for addressing this question by modeling the rates as identically and independently distributed random variables and using tools from random matrix theory to analyze the steady-state production rate. The analysis reveals a principle of universality: the average protein production rate depends only on the of the set of possible values that the random variable may attain. This explains how total protein production can be stabilized despite the overwhelming stochasticticity underlying cellular processes.

## Introduction

During translation complex molecular machines called ribosomes scan the mRNA codon by codon. The ribosome links amino-acids together in the order specified by the codons to form a polypeptide chain. For each codon, the ribosome “waits” for a transfer RNA (tRNA) molecule that matches and carries the correct amino-acid for incorporating it into the growing polypeptide chain. When the ribosome reaches a stop codon encoding a termination signal, it detaches from the mRNA and the complete amino-acid chain is released.

Several ribosomes may read the same mRNA molecule simultaneously, as this form of “pipelining” increases the protein production rate. The dynamics of ribosome flow along the mRNA strongly affects the production rate and the correct folding of the protein. A ribosome that is stalled for a long time may lead to the formation of a “traffic jam” of ribosomes behind it, and consequently to depletion of the pool of free ribosomes. Mutations affecting the protein translation rates may be associated with various diseases^1^, as well as viral infection efficiency^2^.

As translation is a central metabolic process that consumes most of the energy in the cell^3–7^, cells operate sophisticated regulation mechanisms to avoid and resolve ribosome traffic jams^8–11^. These issues have been studied extensively in recent years using various computational and mathematical models^12^. Another testimony of the importance of ribosome flow is the fact that about half of the currently existing antibiotics target the bacterial ribosome by interfering with translation initiation, elongation, termination and other regulatory mechanisms^13,14^. For example, Aminoglycosides inhibit bacterial protein synthesis by binding to the 30S ribosomal subunit, stabilizing a normal mismatch in codon–anticodon pairing, and leading to mistranslations^15^. Understanding the mechanisms of ribosome-targeting antibiotics and the molecular mechanisms of bacterial resistance is crucial for developing new drugs that can effectively inhibit the synthesis of bacterial proteins^16^.

**Figure 1:**
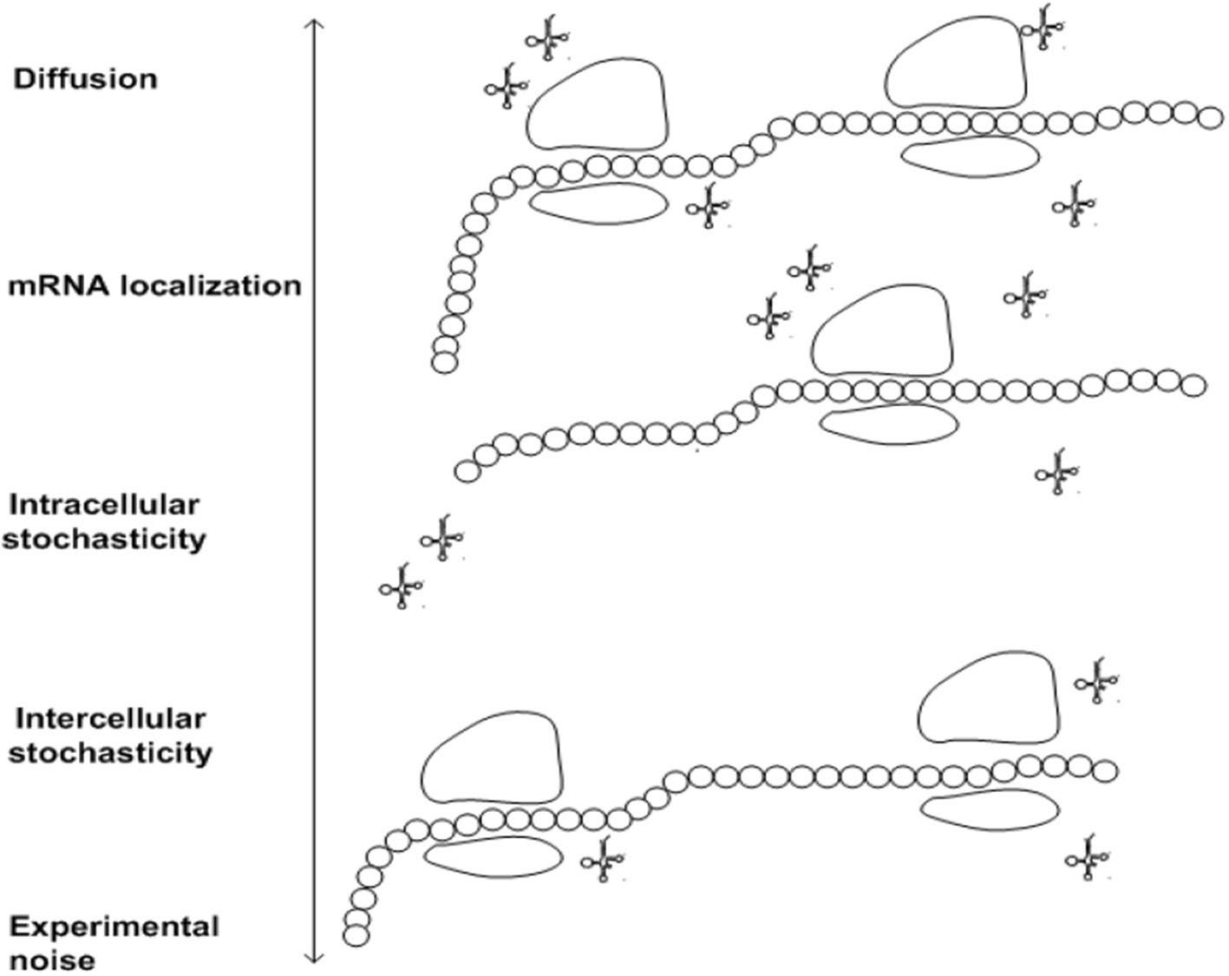
Stochasticity and noise in mRNA translation and its measurements imply that identical mRNAs chains may have different transition rates. The double arrows represent tRNA molecules.

Summarizing, an important problem is to understand the dynamics of ribosome flow along the mRNA, and how it affects the protein production rate. As in many cellular processes, a crucial puzzle is understanding how proper functioning is maintained, and adjusted to the signals that a cell receives and to resource availability, in spite of the large stochasticity in the cell^17,18^. Translation and the measurements of this process are affected by various types of stochasticity (see a review in^19^), as illustrated in Fig. 1. Specifically,

- All the chemical reactions related to the process are of course stochastic, and so are the concentrations of factors like cognate tRNA availability and the resulting translation rates (e.g. during cell cycle), structural accessibility of the $5'$-end to translation factors, the spatial organization of mRNAs inside the cell and the existence of designated “translation factories”^20–23^.
- Different cells in a population are not identical for example in terms of the number of mRNA molecules and ribosomes in the cell and many other aspects^24^.
- It was recently suggested that the ribosomes themselves are not identical^25^.
- The stochastic diffusion of translation substrates play a key role in determining translation rates^26^. The fact that the mRNA molecules of the same gene diffuse (either actively or passively) to different regions in the cell affects their translation properties^27^.
- The experimental approaches for measuring translation introduce various types of noise^28,29^. Thus, the parameters of translation that are inferred from these data are also noisy.
- Processes such as mRNA methylation can affect all aspects of translation^19,30^.
- There are couplings between the translation process and other stochastic gene expression steps^19,31^ such as transcription^32^, mRNA stability^33,34^, and interaction with miRNA^35,36^ and RNA binding proteins^19^.

A recent paper analyzes translation and concludes that “randomness, on average, plays a greater role than any non-random contributions to synthesis time”^37^.

Here, we develop a theoretical approach to analyze translation subject to spatial variation by combining a deterministic computational model, called the ribosome flow model (RFM), with tools from random matrix theory. We model the variation in the initiation, elongation, and exit rates in several copies of the same mRNA by assuming that the rates in the RFM are independent and identically distributed (i.i.d.) random variables, that is, each random variable has the same probability distribution as the others and all are mutually independent. This assumption is of course restrictive, and is needed to obtain our closed-form theatrical results. Yet, it seems to have some empirical justification. For example, away from the ends of the coding sequence the translation rates tend to be independent^38^. In addition, various noise sources (such as NGS noise) tend to be independent along the mRNA. Furthermore, in "Generalizations" section we describe several generalizations where the i.i.d. assumption on the random variables can be relaxed.

We believe that our approach can be used to tackle various levels of stochaticity and uncertainty in translation and its measurements. Our main results (Theorems 1 and 2 below) reveal a new principle of universality: as the length of the mRNA molecule increases the overall steady-state protein production rate converges, with probability one, to a constant value that depends only on the minimal possible value of the random variables. Roughly speaking, this suggests that much of the variability is “filtered out”, and this may explain how the cell overcomes the variations in the many stochastic factors mentioned above.

The next section reviews the RFM and some of its dynamical properties that are relevant in our context. This is followed by our theoretical results. "Generalizations" section describes several generalizations. The final section concludes and describes possible directions for further research.

## Ribosome flow model (RFM)

Mathematical models of the flow of “biological particles” like RNA polymerase, ribosomes, and molecular motors are becoming increasingly important, as powerful experimental techniques provide rich data on the dynamics of such machines inside the cell^39–41^, sometimes in real-time^42^. Computational models are particularly important in fields like synthetic biology and biotechnology, as they can provide qualitative and quantitative testifiable predictions on the effects of various manipulations of the genetic machinery. They are also helpful for understanding the evolution of cells and their biophysics^43^.

The standard computational model for the flow of biological particles is the *asymmetric simple exclusion process* (ASEP)^44–48^. This is a fundamental model from nonequilibrium statistical mechanics describing particles that hop randomly from a site to a neighboring site along an ordered (usually 1D) lattice. Each site may be either free or occupied by a single particle, and hops may take place only to a free target site, representing the fact that the particles have volume and cannot overtake one another. This simple exclusion principle generates an indirect coupling between the moving particles. The motion is assumed to be directionally asymmetric, i.e., there is some preferred direction of motion. In the *totally asymmetric simple exclusion process* (TASEP) the motion is unidirectional.

TASEP and its variants have been used extensively to model and analyze natural and artificial processes including ribosome flow, vehicular and pedestrian traffic, molecular motor traffic, the movement of ants along a trail, and more^43,49,50^. However, due to the intricate indirect interactions between the hopping particles, analysis of TASEP is difficult, and closed-form results exist only in some special cases^51,52^.

**Figure 2:**
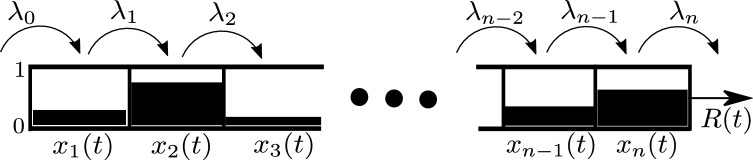
Unidirectional flow along an *n* site RFM. State variable $x_i(t)\in [0,1]$ represents the normalized density at site *i* at time *t*. The parameter $\lambda _i>0$ controls the transition rate from site *i* to site $i+1$, with $\lambda _0$ [$\lambda _n$] controlling the initiation [termination] rate. $R(t)$ is the output rate from the chain at time *t*.

The RFM^53^ is a deterministic, nonlinear, continuous-time ODE model that can be derived via a dynamic mean-field approximation of TASEP^54^. It is amenable to rigorous analysis using tools from systems and control theory. The RFM includes *n* sites ordered along a 1D chain. The normalized density (or occupancy level) of site *i* at time *t* is described by a state variable $x_i(t)$ that takes values in the interval [0, 1], where $x_i(t)=0$ [$x_i(t)=1$] represents that site *i* is completely free [full] at time *t*. The transition between sites *i* and site $i+1$ is regulated by a parameter $\lambda _i>0$. In particular, $\lambda _0$ [$\lambda _n$] controls the initiation [termination] rate into [from] the chain. The rate at which particles exit the chain at time *t* is a scalar denoted by *R*(*t*) (see Fig. 2).

When modeling the flow of biological machines like ribosomes the chain models an mRNA molecule coarse-grained into *n* sites. Each site is a codon or a group of consecutive codons, and *R* (*t*) is the rate at which ribosomes detach from the mRNA, i.e. the protein production rate. The values of the $\lambda _i$s encapsulate many biophysical properties like the number of available free ribosomes, the nucleotide context surrounding initiation codons, the codon compositions in each site and the corresponding tRNA availability, and so on^53,55,56^. Note that these factors may vary in different locations inside the cell.

The dynamics of the RFM is described by *n* nonlinear first-order ordinary differential equations:

1 $$\begin{aligned} {\dot{x}}_i=\lambda _{i-1}x_{i-1}(1-x_i)-\lambda _i x_i(1-x_{i+1}),\quad i=1,\dots ,n, \end{aligned}$$

where we define $x_0(t): = 1$ and $x_{n+1}(t) : = 0$. Every $x_i$ is dimensionless, and every rate $\lambda _i$ has units of $1/{\text {time}}$. Eq. (1) can be explained as follows. The flow of particles from site *i* to site $i+1$ is $\lambda _{i} x_{i}(t) (1 - x_{i+1}(t) )$. This flow is proportional to $x_i(t)$, i.e. it increases with the occupancy level at site *i*, and to $(1-x_{i+1}(t))$, i.e. it decreases as site $i+1$ becomes fuller. This is a “soft” version of the simple exclusion principle. The maximal possible flow from site *i* to site $i+1$ is the transition rate $\lambda _i$. Eq. (1) is thus a simple balance law: the change in the density $x_i$ equals the flow entering site *i* from site $i-1$, minus the flow exiting from site *i* to site $i+1$. The output rate from the last site at time *t* is ${R} (t):=\lambda _n x_n(t)$.

An important property of the RFM (inherited from TASEP) is that it can be used to model and analyze the formation of “traffic jams” of particles along the chain. It was shown that traffic jams during translation are common phenomena even under standard conditions^57^. Indeed, suppose that there exists an index *j* such that $\lambda _j$ is much smaller than all the other rates. Then Eq. (1) gives

$$\begin{aligned} {\dot{x}}_j&= \lambda _{j-1}x_{j-1}(1-x_j)-\lambda _j x_j(1-x_{j+1})\\&\approx \lambda _{j-1}x_{j-1}(1-x_j), \end{aligned}$$

this term is positive when $x\in (0,1)^n$, so we can expect site *j* to fill up, i.e. $x_j(t) \rightarrow 1$. Now using Eq. (1) again gives

$$\begin{aligned} {\dot{x}}_{j-1}&= \lambda _{j-2}x_{j-2}(1-x_{j-1})-\lambda _{j-1} x_{j-1} (1-x_{j})\\&\approx \lambda _{j-2}x_{j-2}(1-x_{j-1}) , \end{aligned}$$

suggesting that site $j-1$ will also fill up. In this way, a traffic jam of particles is formed “behind” the bottleneck rate $\lambda _j$.

Note that if $\lambda _j=0$ for some index *j* then the RFM splits into two separate chains, so we always assume that $\lambda _j>0$ for all $j \in \{0,\dots ,n\}$.

The asymptotic behavior of the RFM has been analyzed using tools from contraction theory^58^, the theory of cooperative dynamical systems^59^, continued fractions and Perron-Frobenius theory^60^. We briefly review some of these results that are required later on.

### Dynamical properties of the RFM

Let *x*(*t*, *a*) denote the solution of the RFM at time $t \ge 0$ for the initial condition $x(0)=a$. Since the state-variables correspond to normalized occupancy levels, we always assume that *a* belongs to the closed *n*-dimensional unit cube:

$$\begin{aligned}{}[0,1]^n:=\{x \in {{\mathbb {R}}}^n: x_i \in [0,1] ,\; i=1,\dots ,n\}. \end{aligned}$$

Let $(0,1)^n$ denote the interior of $[0,1]^n$.

It was shown in^59^ (see also^58^) that there exists a unique $e=e(\lambda _0,\dots ,\lambda _n)\in (0,1)^n$ such that for any $a \in [0,1]^n$ the solution satisfies $x(t,a)\in (0,1)^n$ for all $t > 0$ and

$$\begin{aligned} \lim _{t\rightarrow \infty }x(t,a)=e. \end{aligned}$$

In other words, every state-variable remains well-defined in the sense that it always takes values in [0, 1], and the state converges to a unique steady-state that depends on the $\lambda _i$s, but not on the initial condition. At the steady-state, the flows into and out of each site are equal, and thus the density in the site remains constant. Note that the production rate ${R}(t)=\lambda _n x_n(t)$ converges to the steady-state value ${R} :=\lambda _n e_n$, as $t\rightarrow \infty$. The rate of convergence to the steady-state *e* is exponential^61^.

At the steady-state, the left hand-side of Eq. (1) is zero, and this gives

2 $$\begin{aligned} \lambda _i e_i(1-e_{i+1}) = {R} ,\quad i=0,1,\dots ,n, \end{aligned}$$

where we define $e_0 :=1$ and $e_{n+1} :=0$. In other words, at the steady-state the flow into and out of each site are equal to *R*.

Solving the set of non-linear equations in Eq. (2) is not trivial. Fortunately, there exists a better representation of the mapping from the rates $\lambda _0,\dots ,\lambda _n$ to the steady-state $e_1,\dots ,e_n$. Let ${{\mathbb {R}}}^k_{>0}$ denote the set of *k*-dimensional vectors with all entries positive. Define the $(n+2)\times (n+2)$ tridiagonal matrix

3 $$\begin{aligned} {T}_n := \begin{bmatrix} 0 &{} \lambda _0^{-1/2} &{} 0 &{} \dots &{}0&{}0 \\ \lambda _0^{-1/2} &{} 0 &{} \lambda _1^{-1/2} &{} \dots &{}0&{}0 \\ 0&{} \lambda _1^{-1/2} &{} 0 &{} \dots &{}0&{}0 \\ &{} &{}&{}\vdots \\ 0&{} 0 &{} 0 &{} \dots &{} 0&{} \lambda _{n }^{-1/2} \\ 0&{} 0 &{} 0 &{} \dots &{} \lambda _{n }^{-1/2} &{} 0 \end{bmatrix}. \end{aligned}$$

This is a symmetric matrix, so all its eigenvalues are real. Since every entry of ${T}_n$ is non-negative and ${T}_n$ is irreducible, it admits a simple maximal eigenvalue $\sigma >0$ (called the Perron eigenvalue or Perron root of ${T}_n$), and a corresponding eigenvector $\zeta \in {{\mathbb {R}}}^{n+2}_{>0}$ (the Perron eigenvector) that is unique (up to scaling)^62^.

Given an RFM with dimension *n* and rates $\lambda _0,\dots ,\lambda _n$, let ${T}_n$ be the matrix defined in Eq. (3). It was shown in^63^ that then

4 $$\begin{aligned} {R} =\sigma ^{-2} \text { and } e_i =\lambda _i^{-1/2}\sigma ^{-1}\frac{\zeta _{i+2}}{\zeta _{i+1}}, \quad i=1,\dots ,n. \end{aligned}$$

In other words, the steady-state density and production rate in the RFM can be directly obtained from the spectral properties of ${T}_n$. In particular, this makes it possible to determine *R* and *e* even for very large chains using efficient and numerically stable algorithms for computing the Perron eigenvalue and eigenvector of a tridiagonal matrix.

The spectral representation has several useful theoretical implications. It implies that that ${R} ={R} (\lambda _0,\dots ,\lambda _n)$ is a *strictly concave function* on ${{\mathbb {R}}}^{n+1}_{>0}$. Thus, the problem of maximizing  *R* under an upper bound on the sum of the rates always admits a unique solution^63^.

Also, the spectral representation implies that the sensitivity of the steady-state w.r.t. a perturbation in the rates becomes an eigenvalue sensitivity problem. Known results on the sensitivity of the Perron root^64^ imply that

5 $$\begin{aligned} \frac{\partial }{\partial \lambda _i} {R}&=\frac{2}{\sigma ^3 \lambda _i^{3/2} \zeta '\zeta } \zeta _{i+1} \zeta _{i+2} , \quad i=0,\dots ,n, \end{aligned}$$

where $\zeta '$ denotes the transpose of the vector $\zeta$. It follows in particular that $\frac{\partial }{\partial \lambda _i} {R} >0$ for all *i*, that is, an increase in any of the transition rates yields an increase in the steady-state production rate^60^.

The RFM has been used to analyze various properties of translation. These include mRNA circularization and ribosome cycling^65^, maximizing the steady-state production rate under a constraint on the rates^63,66^, optimal down regulation of translation^67^, and the effect of ribosome drop off on the production rate^68^. More recent work focused on coupled networks of mRNA molecules. The coupling may be due to competition for shared resources like the finite pool of free ribosomes^69,70^, or due to the effect of the proteins produced on the promoters of other mRNAs^71^. Several variations and generalizations of the RFM have also been suggested and analyzed^54,68,72–75^.

Several studies compared predictions of the RFM with biological measurements. For example, protein levels and ribosome densities in translation^53^, and RNAP densities in transcription^76^. The results demonstrate high correlation between gene expression measurements and the RFM predictions.

All previous works on the RFM assumed that the transition rates $\lambda _i$ are deterministic. Here, we analyze for the first time the case where the rates are random variables. This may model for example the parallel translation of copies of the same mRNA molecule in different locations inside the cell. The variance of factors like tRNA abundance in these different locations implies that each mRNA is translated with different rates. It is natural to model this variability using tools from probability theory. For example, Ref.^77^ showed that the distribution of read counts related to a codon in ribo-seq experiments can be approximated using an exponentially modified Gaussian.

Our results analyze the average steady-state production rate given the random transition rates. Note that this provides a global picture of protein production in the cell, rather than the local production in any single chain. For example, when “drawing” the rates from a given distribution, one rate may turn out to be much smaller than the others and this will generate a traffic jam in the corresponding chain. However, our analysis does not consider any specific chain, but the average steady-state production rate on all the chains drawn according to the distribution of the i.i.d. rates.

The following section describes our main results on translation with random rates.

## Main results

Assume that the RFM rates are not constant, but rather are random variables with some known distribution supported over ${{\mathbb {R}}}_{\ge \delta }:= \{x\in {{\mathbb {R}}}: x\ge \delta \}$, where $\delta >0$. What will the statistical properties of the resulting protein production rate be? In the context of the spectral representation given in Eq. (3), this amounts to the following question: given the distributions of the random variables $\{\lambda _i\}_{i=0}^n$, what are the statistical properties of the maximal eigenvalue $\sigma$ of the random matrix ${\mathsf {T}}_n$?

Recall that a random variable ${\mathsf {X}}$ is called *essentially bounded* if there exists $0\le {b}<\infty$ such that ${{\mathbb {P}}}\left[ \left| {\mathsf {X}}\right| \le b\right] =1$, and then the $L_\infty$ norm of ${\mathsf {X}}$ is

$$\begin{aligned} \Vert {\mathsf {X}}\Vert _\infty : = \inf _{ {b}\ge 0} \left\{ {{\mathbb {P}}}\left[ \left| {\mathsf {X}}\right| \le {b}\right] =1\right\} . \end{aligned}$$

Roughly speaking, this is the maximal value that ${\mathsf {X}}$ can attain. Clearly, bounded random variables is the relevant case in any biological model. In particular, if ${\mathsf {X}}$ is supported over ${{\mathbb {R}}}_{\ge \delta }$, with $\delta >0$, then the random variable defined by ${\mathsf {W}}:= {\mathsf {X}}^{-1/2}$ is essentially bounded and $||{\mathsf {W}} ||_\infty \le \delta ^{-1/2}$.

We can now state our main results. To increase readability, all the proofs are placed in the final section of this paper. To emphasize that now the production rate is a random variable, and that it depends on the length of the chain, from hereon we use ${\mathsf {R}}_n$ to denote the production rate in the *n*-site RFM.

### **Theorem 1**

*Suppose that every rate* $\lambda _0,\dots ,\lambda _n$ *in the RFM is drawn independently according to the distribution of an random variable* ${\mathsf {X}}$ *that is supported on* ${\mathbb {R}}_{\ge \delta }$, *with* $\delta >0$. *Then as* $n\rightarrow \infty$, *the maximal eigenvalue of the matrix* ${\mathsf {T}}_n$ *converges to* $2 ||{\mathsf {X}}^{-1/2}||_\infty$ *with probability one, and the steady-state production rate* ${\mathsf {R}}_n$ in *the RFM converges to*

6 $$\begin{aligned} (2 ||{\mathsf {X}}^{-1/2}||_\infty )^{-2}, \end{aligned}$$

*with probability one.*

This result may explain how proper functioning is maintained in spite of significant variability in the rates: the steady-state production rate always converges to the value in Eq. (6), that depends only on $||{\mathsf {X}}^{-1/2}||_\infty$. This also implies a form of universality with respect to the noises and uncertainties: the exact details of the distribution of ${\mathsf {X}}$ are not relevant, but only the single value $||{\mathsf {X}}^{-1/2}||_\infty$.

In general, the convergence to the values in Theorem 1 as *n* increases is slow, and computer simulations may require *n* values that exhaust the computer’s memory before we are close to the theoretical values. The next example demonstrates a case where the convergence is relatively fast.

### **Example 1**

Recall that the probability density function of the half-normal distribution with parameters $(\mu ,\sigma )$ is

$$\begin{aligned} f(x)= {\left\{ \begin{array}{ll} \sqrt{ \frac{2}{\pi \sigma ^2}} \exp ( -\frac{1}{2} (\frac{x-\mu }{\sigma } )^2 ), &{}\quad x\ge \mu ,\\ 0, &{}\quad \text {otherwise}. \end{array}\right. } \end{aligned}$$

**Figure 3:**
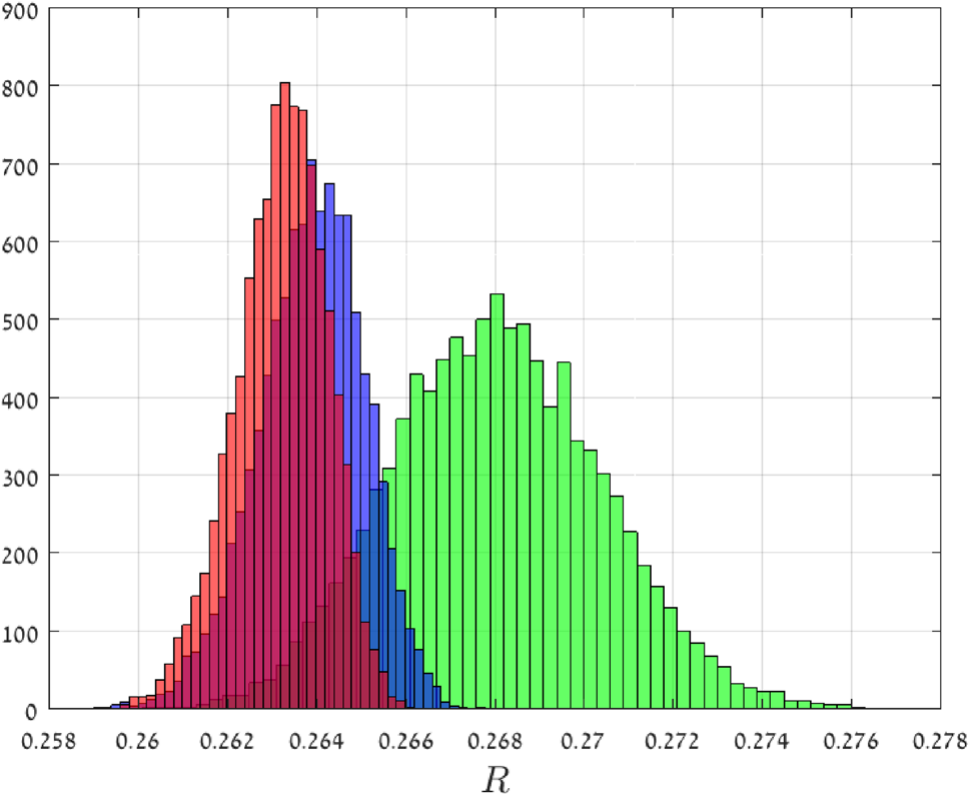
Histograms of 10, 000 ${\mathsf {R}}_n$ values in Example 1 for $n=50$ (green), $n=500$ (blue), and $n=1000$ (red). The theory predicts that as $n\rightarrow \infty$, ${\mathsf {R}}_n$ converges to 1/4 with probability one.

This may be interpreted as a kind of normal distribution, but with support over $[\mu ,\infty )$ only. Suppose that ${\mathsf {X}}$ has this distribution with parameters $( \mu =1,\sigma = 0.1 )$. Note that ${\mathsf {X}}^{-1/2}$ has support (0, 1], so $||{\mathsf {X}}^{-1/2}||_\infty =1$. In this case, Theorem 1 implies that ${\mathsf {R}}_n$ converges with probability one to 1/4 as *n* goes to infinity. For $n\in \{50,500,1000\}$, we numerically computed ${\mathsf {R}}_n$ using the spectral representation for 10, 000 random matrices. Figure 3 depicts a histogram of the results. It may be seen that as *n* increases the histogram becomes “sharper” and its center converges towards 1/4, as expected.

Theorem 1 does not provide any information on the rate of convergence to the limiting value of ${\mathsf {R}}_n$. This is important as in practice *n* is always finite. The next result addresses this issue. For $\epsilon >0$, let

$$\begin{aligned} a(\epsilon ):= {{\mathbb {P}}}\left( {\mathsf {X}}^{-1/2} \ge \Vert {\mathsf {X}}^{-1/2}\Vert _\infty -\epsilon \right) . \end{aligned}$$

Note that $a(\epsilon ) \in (0,1]$. Intuitively speaking, $a(\epsilon )$ is the probability that ${\mathsf {X}}^{-1/2}$ falls in the range $[\Vert {\mathsf {X}}^{-1/2}\Vert _\infty -\epsilon ,\Vert {\mathsf {X}}^{-1/2}\Vert _\infty ]$.

### **Theorem 2**

*Suppose that every rate* $\lambda _0,\dots ,\lambda _n$ *in the RFM is drawn independently according to the distribution of an random variable* ${\mathsf {X}}$ *that is supported on* ${\mathbb {R}}_{\ge \delta }$, *with* $\delta >0$. *Pick two sequences of positive integers* $n_1<n_2<\dots$ *and* $k_1<k_2<\dots$, *with* $k_i<n_i$ *for all* *i*, *and a decreasing sequence of positive scalars* $\epsilon _i$, *with* $\epsilon _i \rightarrow 0$. *Then for any* *i* *the steady-state production rate* ${\mathsf {R}}_{n_i}$ *in an RFM with* $n_i$ *sites satisfies*

7 $$\begin{aligned} (2\Vert {{\mathsf {X}}^{{\mathsf {-1/2}}}}\Vert _\infty )^{-2}\le {\mathsf {R}}_{n_i}&\le (2\Vert {{\mathsf {X}}^{{\mathsf {-1/2}}}}\Vert _\infty )^{-2} \left( 1+O(\epsilon _i+k_i^{-2}) \right) , \end{aligned}$$

*with probability at least*

8 $$\begin{aligned} 1-\exp \left( - { \left\lfloor \frac{n_i-1}{k_i} \right\rfloor } ( a(\epsilon _i))^{k_i } \right) . \end{aligned}$$

Note that if we choose the sequences such that

9 $$\begin{aligned} \frac{n_i}{k_i} (a(\epsilon _i))^{k_i} \rightarrow \infty , \end{aligned}$$

and take $i \rightarrow \infty$ then Theorem 2 yields Theorem 1. Yet, we state and prove both results separately in the interest of readability.

### **Example 2**

Suppose that ${\mathsf {X}}$ has a uniform distribution over an interval $[\delta ,\gamma ]$ with $0< \delta <\gamma$. From here on we assume for simplicity that $\delta =1$ and $\gamma =2$. Then for any $\epsilon >0$ sufficiently small, we have

$$\begin{aligned} a(\epsilon )&= {{\mathbb {P}}}\left( {\mathsf {X}}^{-1/2} \ge 1-\epsilon \right) \\&= {{\mathbb {P}}}\left( {\mathsf {X}} \le (1-\epsilon )^{-2} \right) \\&=2\epsilon +o(\epsilon ). \end{aligned}$$

Fix $d\in (0,1)$ and take $\epsilon _i = n_i^{(d-1)/k_i}$. Then the condition in Eq. (9) becomes

$$\begin{aligned} \frac{n_i^d}{k_i} \rightarrow \infty \end{aligned}$$

and this will hold if $k_i$ does not increase too quickly. We can write $\epsilon _i$ as

$$\begin{aligned} \epsilon _i=\exp ( (d-1)\log (n_i)/k_i ), \end{aligned}$$

so to guarantee that $\epsilon _i \rightarrow 0$, we take $k_i=(\log (n_i))^c$, with $c\in (0,1)$, and then Eq. (9) indeed holds. Theorem 2 implies that

$$\begin{aligned}&(2\Vert {{\mathsf {X}}^{{\mathsf {-1/2}}}}\Vert _\infty )^{-2}\le {\mathsf {R}}_{n_i} \le (2\Vert {{\mathsf {X}}^{{\mathsf {-1/2}}}}\Vert _\infty )^{-2} \left( 1+O( \max \{\exp ( (d-1) (\log (n_i) )^{1-c} ) , (\log (n_i))^{-2c} \} ) \right) , \end{aligned}$$

with probability at least

10 $$\begin{aligned} 1-\exp \left( \frac{-n_i^d}{(\log (n_i))^c} \right) . \end{aligned}$$

### **Example 3**

As in Example 1, consider the case where ${\mathsf {X}}$ is half-normal with parameters $(\mu ,\sigma )$, where $\mu >0$. Then $\Vert {\mathsf {X}}^{-1/2}\Vert _\infty = \mu ^{-1/2}$, so

$$\begin{aligned} a(\epsilon )&= {{\mathbb {P}}}\left( {\mathsf {X}} ^{-1/2} \ge \mu ^{-1/2}-\epsilon \right) \\&= {{\mathbb {P}}}\left( {\mathsf {X}}\le z \right) , \end{aligned}$$

where $$z := (\mu ^{-1/2}-\epsilon )^{-2}$$. Thus,

$$\begin{aligned} a(\epsilon )&= \sqrt{\frac{2}{\pi \sigma ^2}}\int _{\mu }^{z}e^{-\frac{(x-\mu )^2}{2\sigma ^2}}\mathrm {d}x\\&= \frac{2}{ \sqrt{\pi }}\int _{0}^{\frac{ z-\mu }{\sqrt{2\sigma ^2}}}e^{-x^2}\mathrm {d}x. \end{aligned}$$

It is not difficult to show that this implies that

11 $$\begin{aligned} a(\epsilon ) = c(\mu ,\sigma ) \epsilon +o(\epsilon ) , \end{aligned}$$

where $c(\mu ,\sigma ) : = 2 \sqrt{ \frac{2}{\pi \sigma ^2}} \mu ^{3/2}$. To satisfy Eq. (9), fix $p\in (0,1)$ and choose $\epsilon _i$ such that $(c \epsilon _i)^{k_i} = n_i^{p-1}$. This implies that

12 $$\begin{aligned} \epsilon _i = \frac{1}{c } \exp \left( \frac{p-1}{k_i}\log (n_i)\right) . \end{aligned}$$

Now, pick $q\in (0,1)$ and take $k_i = (\log ( n_i))^q$. Then Eq. (9) holds, and

13 $$\begin{aligned} \epsilon _i = \frac{1}{c } \exp \left( (p-1) ( \log (n_i) ) ^{1-q}\right) . \end{aligned}$$

Theorem 2 implies that for any $p,q\in (0,1)$, we have

$$\begin{aligned} \frac{\mu }{4}\le {\mathsf {R}}_{n_i} \le \frac{\mu }{4} +O\left( \max \left\{ \frac{1}{c } \exp \left( (p-1) ( \log (n_i) ) ^{1-q}\right) , (\log (n_i))^{-2q} \right\} \right) , \end{aligned}$$

with probability at least

$$\begin{aligned} 1-\exp \left( \frac{-n_i^p}{ (\log (n_i))^q } \right) . \end{aligned}$$

This shows that ${\mathsf {R}}_{n_i}$ “is close” to $\mu /4$, and provides an explicit expression for the rate of convergence to $\mu /4$.

## Generalizations

The assumption that all the rates are i.i.d. random variables allows to derive the general theoretical results in Theorems 1 and 2 above. However, this assumption is restrictive. In this section, we describe several cases where we allow more relaxed assumptions on these rates. Our first generalization considers the case where the random variables might be non-identical, but all share the same support. In the second generalization, we allow an increasing (but small compared to *n*) number of random variables to have a different support from the majority of the other random variables. In these two cases we show that the production rate converges to the same value as in Theorem 1.

We then turn to investigate the most general case, where the rates are arbitrary but bounded, and in this case provide lower and upper bounds on the production rate.

Analysis of the proofs of Theorems 1 and 2 shows that our results remain valid even if each rate $\lambda _i$ is drawn from the distribution of ${\mathsf {X}}_i$, which are not necessarily identically distributed, but are all independent, supported on the positive semi-axis, and satisfy

14 $$\begin{aligned} ||{\mathsf {X}}_0^{-1/2}||_\infty =\dots =||{\mathsf {X}}_{n}^{-1/2}||_\infty , \end{aligned}$$

namely, they all have the same bound. The next example demonstrates this.

### **Example 4**

**Figure 4:**
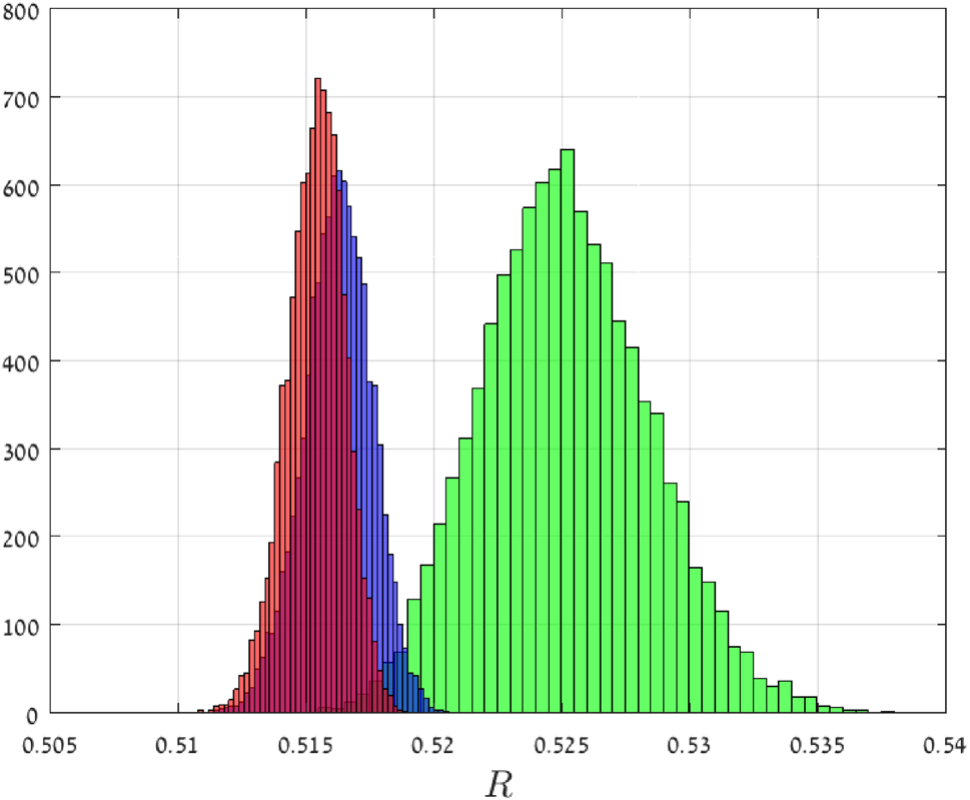
Histograms of 10, 000 ${\mathsf {R}}_n$ values in Example 4 for $n=50$ (green), $n=500$ (blue), and $n=1000$ (red). The theory predicts that as $n\rightarrow \infty$, ${\mathsf {R}}_n$ converges to 1/2 with probability one.

Consider $n+1$ independent random variables with ${\mathsf {X}}_{0},{\mathsf {X}}_{1},\dots ,{\mathsf {X}}_{\frac{n-1}{2}}$ distributed according to the half-normal distribution with parameters $( \mu =2,\sigma = 0.1 )$, and ${\mathsf {X}}_{\frac{n-1}{2}+1},\dots , {\mathsf {X}}_{n}$ distributed according to the uniform distribution on [2, 3]. Note that $||{{\mathsf {X}}_{{\mathsf {i}}}}^{-1/2}||_\infty =2^{-1/2}=1 / \sqrt{2}$, for all $i=0,1,\dots ,n$. Thus, our theory predicts that in this case ${\mathsf {R}}_n$ converges with probability one to $(2/\sqrt{2})^{- 2} = 1/2$ as *n* goes to infinity. For $n\in \{50,500,1000\}$, we numerically computed ${\mathsf {R}}_n$ using the spectral representation for 10, 000 random matrices. Figure 4 depicts a histogram of the results. It may be seen that as *n* increases the histogram becomes “sharper” and its center converges towards 1/2, as expected.

Our second generalization considers the case where among the $n+1$ random rates there are *d* rates drawn from the distributions of the random variables ${\mathsf {Y}}_1,\dots , {\mathsf {Y}}_d$, that might have some different distributions; they do not have to satisfy the uniform support condition in Eq. (14), and they might be dependent. Here $d=d(n)$ is an integer that is allowed to grow with *n*, but at a slower rate than *n*. We assume that the rates modeled by these random variable are larger those rates modeled by the other $n+1-d$ random variables (see Eq. (15) below).

### **Theorem 3**

*Let* $d=d(n)>0$ *be an integer such that* $\lim _{n\rightarrow \infty } \frac{d(n)}{n} =0$. *Let* $\{{\mathsf {X}}_i\}_{i=0}^{n-d}$ *be a set of* $(n+1-d)$ *independent random variables, supported on* ${\mathbb {R}}_{\ge \delta }$, *with* $\delta >0$, *and satisfying*

$$\begin{aligned} ||{\mathsf {X}}_0^{-1/2}||_\infty =\dots =||{\mathsf {X}}_{n-d}^{-1/2}||_\infty . \end{aligned}$$

*Also, let* $\{{\mathsf {Y}}_i\}_{i=1}^d$ *be a set of* *d* *random variables supported on the positive semi-axis, and satisfy*

15 $$\begin{aligned} ||{\mathsf {Y}}_j^{-1/2}||_\infty \le \delta ^{-1/2}, \quad j=1,\dots ,d. \end{aligned}$$

*Fix* $\epsilon >0$ *and a positive integer* *k*. *Denote the concatenation of* $\{{\mathsf {Y}}_i\}_{i=1}^d$ *and* $\{{\mathsf {X}}_i\}_{i=0}^{n-d }$ by ${\mathsf {Z}}$, *namely,* ${\mathsf {Z}} = ({\mathsf {Y}}_1,{\mathsf {Y}}_2,\ldots ,{\mathsf {Y}}_d,{\mathsf {X}}_0,{\mathsf {X}}_1,\ldots ,{\mathsf {X}}_{n-d })$. *Let* ${\mathcal {S}}^{n+1}$ *denote the set of permutations on* $\{1,\dots ,n+1\}$. *Fix a permutation* $\pi \in {\mathcal {S}}^{n+1}$, *and let* ${\mathsf {Z}}^\pi \triangleq \pi \circ {\mathsf {Z}}$. *Suppose that every rate* $\lambda _i$ *in the RFM is drawn independently according to the distribution of the random variables in* ${\mathsf {Z}}^\pi _i$. *Then as* $n\rightarrow \infty$, *the steady-state production rate* ${\mathsf {R}}_n$ *in the RFM converges to*

16 $$\begin{aligned} (2 ||{\mathsf {X}}_0^{-1/2}||_\infty )^{-2}, \end{aligned}$$

*with probability one.*

In other words, even in the presence of the “interfering” ${\mathsf {Y}}_i$’s the theoretical result remains unchanged. The next example demonstrates Theorem 3.

### **Example 5**

**Figure 5:**
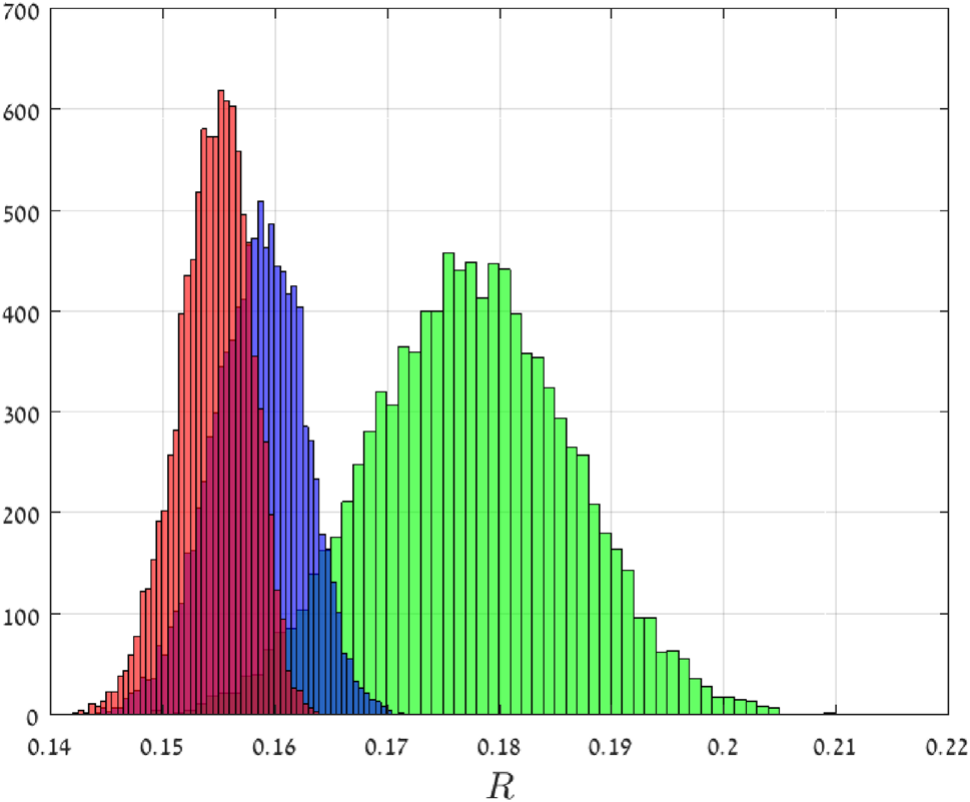
Histograms of 10, 000 ${\mathsf {R}}_n$ values in Example 5 for $n=50$ (green), $n=500$ (blue), and $n=1500$ (red). The theory predicts that as $n\rightarrow \infty$, ${\mathsf {R}}_n$ converges to 1/8 with probability one.

Consider the case where $d(n)=\sqrt{n}$. Let ${\mathsf {X}}_0,\dots ,{\mathsf {X}}_{n-d}$ be i.i.d. random variables distributed according to the uniform distribution on [1/2, 1], and let Let ${\mathsf {Y}}_1,\dots ,{\mathsf {Y}}_{d}$ be i.i.d. random variables distributed according to the uniform distribution on [15, 20]. We draw the rates according to the vector ${\mathsf {Z}}^{\pi }$, with $\pi$ a random permutation (implemented using the Matlab command *randperm*). Our theory predicts that in this case ${\mathsf {R}}_n$ converges with probability one to $(2||{\mathsf {X}}_i^{-1/2}||_\infty )^{-2} =(2 \sqrt{2})^{-2}=1/8$ as *n* goes to infinity. For $n\in \{50,500,1500\}$, we numerically computed ${\mathsf {R}}_n$ using the spectral representation for 10, 000 random matrices. Figure 5 depicts a histogram of the results. It can be seen that the ${\mathsf {R}}_n$ converges with probability one to a limiting value, despite the “interfering” ${\mathsf {Y}}_i$ random variables.

Our last and most general result considers the case where the random variables are arbitrary but bounded. In particular, they do not necessarily have to be independent or identical. We use the notation ${\mathcal {I}}_k^{p}$ to denote the set of all possible *k* consecutive integers from the set $\{1,2,\dots ,p\}$. For example,

$$\begin{aligned} {\mathcal {I}}_2^3 = \{ \{1,2\}, \{1,3\}, \{2,3\} \}. \end{aligned}$$

### **Theorem 4**

*Suppose that every rate* $\lambda _i$ *in the RFM is drawn according to the distribution of a random variable* ${\mathsf {X}}_i$ *that is supported on* ${\mathbb {R}}_{\ge \delta _i}$, *with* $\delta _i>0$, *for* $0\le i\le n$. *Then the steady-state production rate* ${\mathsf {R}}_n$ *in the* *n*-*site RFM satisfies*

17 $$\begin{aligned} \left[ \max _{i=1,\ldots ,n}{\mathsf {X}}_{i-1}^{-1/2}+{\mathsf {X}}_i^{-1/2}\right] ^{-2}\le {\mathsf {R}}_n&\le \left[ 2\max _{1\le k\le n+1}\cos \left( \frac{\pi }{k+2}\right) \max _{{\mathsf {I}}_k\in {\mathcal {I}}_k^{n+1}}\min _{i\in {\mathsf {I}}_k}{\mathsf {X}}_i^{-1/2}\right] ^{-2}, \end{aligned}$$

*with probability one.*

Contrary to our previous analytical results, in this case the steady-state production rate will not necessarily converge to a deterministic value, but rather we show that it is bounded above and below by two random quantities. However, it can be shown that when the random variables are i.i.d. then both bounds converge to $(2||{\mathsf {X}}_0^{-1/2}||_\infty )^{-2}$ as $n\rightarrow \infty$, and in this sense the bounds in Theorem 4 are tight.

## Discussion

Cellular systems are inherently noisy, and it is natural to speculate that they were optimized by evolution to function properly, or even take advantage, of stochastic fluctuations.

Many studies analyzed the fluctuations in protein production due to both extrinsic and intrinsic noise (see, e.g.^18,78–82^). Here, we derived a new approach, based on random matrix theory, for analyzing the average protein production rate from multiple copies of the same mRNA that are affected by variations in the translation rates due, for example, to the different spatial location of these mRNAs inside the cell. Our approach can also deal with experimental noise.

Our results have both a theoretical and a practical value. We show that given one parameter value $\delta$ from the i.i.d. distribution allows to determine the steady-state average production rate. The production rate is thus agnostic to many other details underlying the distribution e.g. it’s mean, variance, etc. This may explain how steady-state production is maintained despite the considerable stochasticity in the cell. This theoretical result holds regardless of whether one can actually determine the value $\delta$ or not.

Our approach can also deal with phenomena that is not directly captured by the RFM, if its affects can be modeled as a stochastic perturbation of the transition rates. Examples may include experimental noise, methylation, and interaction with miRNA. In particular, methylation affects one nucleotide/codon, and miRNA affects a sequence of up to 7 codons.

It is important to note that our results hold for many possible distributions of the translation rates. For example, it was suggested that decoding rates distributions are similar to an exponential modified Gaussian or log normal distributions^77,83^.

Currently, it is challenging to estimate the distribution of transition rates (and thus the bound on the support $\delta$). Indeed, approaches such as ribo-seq plot averages over all mRNA molecules and all cells in a certain population/sample. It is also difficult to estimate the protein translation rate. Usually, the measured quantity is protein level, but this depends not only on translation, but also on the rate of transcription, and mRNA and protein dilution and decay^79^. Thus, in this respect, the theory in the paper precedes biological measurement capabilities. Our results however may indicate general principles that can be tested experimentally. For example, the analysis suggests that as the length of the mRNA increases while keeping all its statistical properties such as initiation rate and codon usage identical, the translation rate becomes more uniform.

The RFM, just like TASEP, is a phenomenological model for the flow of interacting particles and thus can be used to model and analyze phenomena like the flow of packets in communication networks^84^, the transfer of a phosphate group through a serial chain of proteins during phosphorelay^75^, and more. The RFM is also closely related to a mathematical model for a disordered linear chain of masses, each coupled to its two nearest neighbors by elastic springs^85^, that was originally analyzed in the seminal work of Dyson^86^. In many of these applications it is natural to assume that the rates are subject to uncertainties or fluctuations and model them as random variables. Then the results here can be immediately applied.

We believe that the approach described here can be generalized to other models of intra-cellular phenomena derived from the RFM^75,87^, and thus for analyzing additional aspects of translation and gene expression.

## Proofs

The proofs of our main results are based on analyzing the spectral properties of the matrix ${\mathsf {T}}_n$ in Eq. (3) when the $\lambda _i$s are i.i.d. random variables. The problem that we study here is a classical problem in random matrix theory^88^, yet the matrix ${\mathsf {T}}_n$ is somewhat different from the standard matrices analyzed using the existing theory (e.g. the Wigner matrix). Hence, we provide a self-contained analysis based on combining probabilistic arguments with the Perron-Frobenius theory of matrices with non-negative entries (see e.g.^62^, Ch. 8).

### *Proof of Theorem* 1

Recall that the rates $\{\lambda _i\}_{i=0}^{n}$ are drawn independently according to the distribution of a random variable ${\mathsf {X}}$ that is supported on ${\mathbb {R}}_{\ge \delta }$, with $\delta >0$. For simplicity of notation, let ${\mathsf {W}}_i := \lambda _i^{-1/2}$, $i\in \{0,1,\ldots ,n\}$, and note that $\{{\mathsf {W}}_i\}_{i=0}^{n}$ are essentially bounded, i.i.d., and each random variable ${\mathsf {W}}_i$ follows the same distribution of ${\mathsf {X}}^{-1/2}$. In particular, ${\mathsf {W}}_0\equiv {\mathsf {X}}^{-1/2}$. With this definition, Eq. (3) can be written as:

18 $$\begin{aligned} {\mathsf {T}}_n := \begin{pmatrix}0 &{} {\mathsf {W}}_0 &{} &{} &{} \\ {\mathsf {W}}_0 &{} 0 &{} {\mathsf {W}}_1 &{} &{} \\ &{} {\mathsf {W}}_1 &{} 0 &{} \ddots &{} \\ &{} &{} \ddots &{} &{} {\mathsf {W}}_{n} \\ &{} &{} &{} {\mathsf {W}}_{n} &{} 0\end{pmatrix}. \end{aligned}$$

Therefore, ${\mathsf {T}}_n$ is an $(n+2)\times (n+2)$ symmetric tridiagonal matrix, with zeros on its main diagonal, and bounded positive random variables $\{{\mathsf {W}}_i\}_{i=0}^{n}$ on the super- and sub-diagonals.

Since ${\mathsf {T}}_n$ is symmetric, componentwise non-negative, and irreducible, it admits a simple maximal eigenvalue denoted $\lambda _{\max }({\mathsf {T}}_n)$, and $\lambda _{\max }({\mathsf {T}}_n) >0$. Our goal is to understand the asymptotic behavior of $\lambda _{ \max }({\mathsf {T}}_n )$, as $n\rightarrow \infty$. We begin with an auxiliary result that will be used later on.

#### **Proposition 1**

*Suppose that the random variables* $\{{\mathsf {W}}_i\}_{i=0}^{n}$ *are i.i.d. and essentially bounded. Fix* $\epsilon >0$ *and an integer* $1\le k\le n+1$. *Let* ${{\mathcal {K}}}$ *denote the event: there exists an index* $0\le \ell \le n-k+1$ *such that* ${\mathsf {W}}_{\ell },\dots ,{\mathsf {W}}_{\ell +k-1 }\ge \Vert {\mathsf {W}}_0\Vert _\infty -\epsilon$. *Then as* $n\rightarrow \infty$ *the probability of* ${{\mathcal {K}}}$ *converges to one.*

In other words, as $n\rightarrow \infty$ the probability of finding *k* consecutive random variables whose value is at least $\Vert {\mathsf {W}}_0\Vert _\infty -\epsilon$ goes to one.

#### *Proof*

Fix $\epsilon >0$ and a positive integer *k*. Let $s : = \Vert {\mathsf {W}}_0\Vert _\infty -\epsilon$. For any $j\in \{0,\dots ,n-k+1\}$, let ${{{\mathcal {K}}}} (j)$ denote the event: ${\mathsf {W}}_{j },\dots ,{\mathsf {W}}_{j+k -1}\ge s$. Then

$$\begin{aligned} {{\mathbb {P}}}\left( {{{\mathcal {K}}}}\right)&\ge {{\mathbb {P}}}\left( {{{\mathcal {K}}}}(1) \cup {{{\mathcal {K}}}}(k+1) \cup {{{\mathcal {K}}}}(2k+1) \cup \dots \cup {{{\mathcal {K}}}}(pk+1) \right) , \end{aligned}$$

where *p* is the largest integer such that $(p+1)k\le n$. Since the ${\mathsf {W}}_i$s are i.i.d.,

$$\begin{aligned} {{\mathbb {P}}}\left( {{{\mathcal {K}}}}\right)&\ge 1-(1-{{\mathbb {P}}}\left( {{{\mathcal {K}}}}(1) \right) )^{p+1}\\&= 1-(1-({{\mathbb {P}}}\left( {\mathsf {W}}_0 \ge s \right) )^k )^{p+1}. \end{aligned}$$

The probability ${{\mathbb {P}}}\left( {\mathsf {W}}_0 \ge s \right)$ is positive, and when $n\rightarrow \infty$, we have $p\rightarrow \infty$, so ${{\mathbb {P}}}\left( {{{\mathcal {K}}}}\right) \rightarrow 1$. $\square$

The next result invokes Proposition 1 to provide a tight asymptotic lower bound on the maximal eigenvalue of ${\mathsf {T}}_n$.

#### **Proposition 2**

*Suppose that the random variables* $\{{\mathsf {W}}_i\}_{i=0}^{n}$ *are i.i.d. and essentially bounded. Fix* $\epsilon >0$ *and an integer* $1\le k\le n+1$. *Then the probability*

19 $$\begin{aligned} {{\mathbb {P}}}\left( \lambda _{\max }({\mathsf {T}}_n) \ge 2 (\Vert {\mathsf {W}}_0\Vert _\infty -\epsilon ) \cos {\frac{\pi }{k+2}} \right) , \end{aligned}$$

*goes to one as* $n\rightarrow \infty$.

#### *Proof*

Let $s := \Vert {\mathsf {W}}_0\Vert _\infty -\epsilon$. Conditioned on the event ${{{\mathcal {K}}}}$, Proposition 1 implies that there exists an index $\ell$ such that ${\mathsf {W}}_{\ell },\dots ,{\mathsf {W}}_{\ell +k-1 }\ge s$. Assume that $\ell =0$ (the proof in the case $\ell >0$ is very similar). Let ${\mathsf {M}}_{k}$ denote the $(k+1)\times (k+1)$ symmetric tridiagonal matrix:

20 $$\begin{aligned} {\mathsf {M}}_{k} : =\begin{pmatrix}0 &{} 1 &{} &{} &{} \\ 1 &{} 0 &{} 1 &{} &{} \\ &{} 1 &{} 0 &{} \ddots &{} \\ &{} &{} \ddots &{} &{} 1 \\ &{} &{} &{} 1 &{} 0\end{pmatrix}. \end{aligned}$$

Recall that the maximal eigenvalue of this matrix is $\lambda _{{\mathsf {max}}} ({\mathsf {M}}_{k }) =2 \cos {\frac{\pi }{k+2}}$ (see e.g.^89^). Now, let ${\mathsf {P}}_n$ be the matrix obtained by replacing the $(k+1)\times (k+1)$ leading principal minor of ${\mathsf {T}}_n$ by $s {\mathsf {M}}_k$. Note that ${\mathsf {T}}_n \ge {\mathsf {P}}_n$ (where the inequality is componentwise), and thus $\lambda _{\max } ({\mathsf {T}}_n) \ge \lambda _{\max } ({\mathsf {P}}_n)$. By Cauchy’s interlacing theorem, the largest eigenvalue of ${\mathsf {P}}_n$ is larger or equal to the largest eigenvalue of any of its principal minors. Thus,

$$\begin{aligned} \lambda _{\max } ({\mathsf {P}}_n)&\ge \lambda _{\max } (s{\mathsf {M}}_k)\\&\ge 2 s \cos \left( {\frac{\pi }{k+2}}\right) . \end{aligned}$$

and this completes the proof of Proposition 2. $\square$

We can now complete the proof of Theorem 1. Recall that if  *A* is an $n\times n$ symmetric and componentwise non-negative matrix then (see, e.g.^62^, Ch. 8)

21 $$\begin{aligned} \lambda _{{\mathsf {max}}}( {A} ) \le \max _{i\in \{1,\dots ,n\}}\sum _{j=1}^n a_{ij}. \end{aligned}$$

In other words, $\lambda _{{\mathsf {max}}}( {A} )$ is bounded from above by the maximum of the row sums of *A*. As any row of ${\mathsf {T}}_n$ has at most two nonzero elements, Eq. (21) implies that

22 $$\begin{aligned} \lambda _{{\mathsf {max}}}({\mathsf {T}}_n)&\le \max _{i \in \{1,\dots ,n \} } ( {\mathsf {W}}_{i-1}+{\mathsf {W}}_{i} )\nonumber \\&\le 2\max _{i\in \{0,\dots ,n \} }{\mathsf {W}}_i, \end{aligned}$$

with probability one. Combining this with Proposition 2 implies that

23 $$\begin{aligned} 2 (||{\mathsf {W}}_0||_\infty -\epsilon ) \cos \left( {\frac{\pi }{k+2}}\right) \le \lambda _{\max }({\mathsf {T}}_n)\le 2 ||{\mathsf {W}}_0||_\infty , \end{aligned}$$

with probability one. Since this holds for any $\epsilon >0$ and any integer $k>0$, this completes the proof of Theorem 1. $\square$

### *Proof of Theorem* 2

Fix $\epsilon >0$ and an integer $1\le k\le n+1$. Let ${{\bar{a}}}(\epsilon ): = {{\mathbb {P}}}\left( {\mathsf {W}}_0 \ge \Vert {\mathsf {W}}_0\Vert _\infty -\epsilon \right)$. The proofs of Propositions 1 and 2 imply that

24 $$\begin{aligned} \lambda _{{\mathsf {max}}}({\mathsf {T}}_n ) \ge 2 (\Vert {\mathsf {W}}_0\Vert _\infty -\epsilon ) \cos {\frac{\pi }{k+2}}, \end{aligned}$$

with probability ${{\mathbb {P}}}({{\mathcal {K}}})\ge 1-(1-({{\bar{a}}}(\epsilon )) ^k )^{\left\lfloor \frac{n}{k} \right\rfloor }$. Fix $b,c>0$. The trivial bound $1-b<\exp (-b)$ implies that $1-(1-b)^{c } > 1-\exp (-bc)$, and thus,

25 $$\begin{aligned} {{\mathbb {P}}}({{\mathcal {K}}})&\ge 1-(1-({{\bar{a}}}(\epsilon )) ^k )^ {\left\lfloor \frac{n}{k} \right\rfloor }\nonumber \\&\ge 1-\exp \left( - \left\lfloor \frac{n}{k} \right\rfloor ({{\bar{a}}}(\epsilon )) ^k \right) . \end{aligned}$$

Pick two sequences of positive integers $n_1<n_2<\dots$ and $k_1<k_2<\dots$, with $k_i<n_i$ for all *i*, and a decreasing sequence of positive scalars $\epsilon _i$, with $\epsilon _i \rightarrow 0$. Using Eq. (24) we get

$$\begin{aligned} ( \lambda _{\max } ( {\mathsf {T}}_{n_i} ) )^{-2}&\le \left( 2 (\Vert {\mathsf {W}}_0\Vert _\infty -\epsilon _i) \cos {\frac{\pi }{k_i+2}} \right) ^{-2}\\&= (2\Vert {\mathsf {W }}_0\Vert _\infty )^{-2} \left( 1+\frac{\epsilon _i}{\Vert {\mathsf {W }}_0\Vert _\infty } +o(\epsilon _i) \right) \left( \cos {\frac{\pi }{k_i+2}} \right) ^{-2}\\&= (2\Vert {\mathsf {W}}_0\Vert _\infty )^{-2} \left( 1+\frac{\epsilon _i}{\Vert {\mathsf {W}}_0\Vert _\infty } +o(\epsilon _i) \right) \left( 1+\frac{\pi ^2}{(k_i+2)^2} + o(k_i^{-2}) \right) \\&= (2\Vert {\mathsf {W}}_0\Vert _\infty )^{-2} \left( 1+O(\epsilon _i+k_i^{-2})\right) . \end{aligned}$$

Combining this with the spectral representation of the steady-state in the RFM completes the proof of Theorem 2. $\square$

The proofs of Theorems 3 and 4 below are similar to the proof of Theorem 1, and so we only explain the needed modifications in the proof of Theorem 1.

### *Proof of Theorem* 3

The proof of Proposition 1 remains valid due to the fact that $d>0$ is sub-linear in *n*, and we let $n \rightarrow \infty$. Specifically, by the pigeonhole principle it is clear that there must exist a sub-sequence of ${\mathsf {Z}}^{\pi }$, of length at least *n*/*d*, which consists of consecutive ${\mathsf {X}}_i$’s; therefore, we can apply the proof of Proposition 1 on this sub-sequence. In this case, we note that the range of the parameter *p* in the proof of Proposition 1 becomes $(p+1)k\le \left\lfloor n/d \right\rfloor$, and thus as long as $n/d\rightarrow \infty$ we have $p\rightarrow \infty$ as well. Thus, the conclusion of Proposition 2 remains valid. The bound in Eq. (22) also holds, due to the condition in Eq. (15). Thus, Eq. (23) holds, and this completes the proof of Theorem 3. $\square$

### *Proof of Theorem* 4

As in the proof of Theorem 1, define ${\mathsf {W}}_i : = {\mathsf {X}}_i^{-1/2}$, $i\in \{0,1,\ldots ,n\}$. The proof of the upper bound in Theorem 4 is in fact the same as in Eq. (22). Indeed, in Eq. (22) we show that

26 $$\begin{aligned} \lambda _{{\mathsf {max}}}({\mathsf {T}}_n)&\le \max _{i \in \{1,\dots ,n\} } ( {\mathsf {W}}_{i-1}+{\mathsf {W}}_{i}), \end{aligned}$$

which implies the lower bound in Eq. (17). The upper bound in Eq. (17) follows from the same arguments used to obtain Proposition 1. Indeed, for any $1\le k\le n$, let ${\mathsf {I}}_k$ be any set of *k* consecutive indices in $\{0,1,\ldots ,n\}$. Let ${\mathsf {P}}_n$ be the matrix obtained by replacing the $(k+1)\times (k+1)$ principal minor that corresponds to the indices ${\mathsf {I}}_k$ of ${\mathsf {T}}_n$ by ${\mathsf {M}}_k\cdot \min _{i\in {\mathsf {I}}_k}{\mathsf {W}}_i$. Note that ${\mathsf {T}}_n \ge {\mathsf {P}}_n$ (where the inequality is componentwise), and thus $\lambda _{\max } ({\mathsf {T}}_n ) \ge \lambda _{\max } ({\mathsf {P}}_n )$. By Cauchy’s interlacing theorem, the largest eigenvalue of ${\mathsf {P}}_n$ is larger or equal to the largest eigenvalue of any of its principal minors. Thus,

27 $$\begin{aligned} \lambda _{\max } ({\mathsf {P}}_n )&\ge \lambda _{\max } \left( {\mathsf {M}}_k\cdot \min _{i\in {\mathsf {I}}_k}{\mathsf {W}}_i\right) \nonumber \\&\ge 2\min _{i\in {\mathsf {I}}_k}{\mathsf {W}}_i \cdot \cos \left( {\frac{\pi }{k+2}}\right) . \end{aligned}$$

Now, since Eq. (27) holds for any choice of $1\le k\le n$ and ${\mathsf {I}}_k\in {\mathcal {I}}_k^{n+1}$, we can maximize the r.h.s. of Eq. (27) with respect to these assignments, which implies the upper bound in Eq. (17). $\square$
